# Effect of Time and Mixing in Thermal Pretreatment on Faecal Indicator Bacteria Inactivation

**DOI:** 10.3390/ijerph15061225

**Published:** 2018-06-11

**Authors:** Fubin Yin, Hongmin Dong, Bin Shang, Wanqin Zhang

**Affiliations:** Institute of Environment and Sustainable Development in Agriculture, Chinese Academy of Agricultural Sciences, Beijing 100081, China; donghongmin@caas.cn (H.D.); shangbin@caas.cn (B.S.); zhang394982409@126.com (W.Z.)

**Keywords:** faecal sludge, thermal pretreatment, velocity gradient, faecal indicator bacteria inactivation, sludge solubilisation

## Abstract

Faecal indicator bacteria of faecal coliform, *Salmonella* spp., and faecal *Streptococcus* are present at high levels in faecal sludge and affect human health. Mesophilic anaerobic digestion cannot reduce faecal indicator bacteria to meet the standards for biosolids; therefore, the thermal pretreatment of faecal sludge is essential. The main objectives of this research were to evaluate the effect of thermal (70 °C) pretreatment time (20, 40, 60, 80, 100, and 120 min) and mixing velocity gradient (no mixing, 133, 191, and 238 s^−1^) on faecal indicator bacteria inactivation and determine the kinetics of the inactivation. The results showed that mixing has a more positive effect on pretreatment; thermal pretreatment with mixing was able to completely inactivate faecal indicator bacteria within 80 min, and inactivation followed first-order kinetics. In addition, under optimal mixing at a velocity gradient 191 s^−1^, the thermal pretreatment with mixing had a positive effect on the sludge solubilisation. Soluble chemical oxygen demand (SCOD, 71,430 mg L^−1^) and soluble protein (7.96 g L^−1^) were higher than the values obtained with thermal pretreatment without mixing, which were a SCOD value of 63,600 mg L^−1^ and soluble protein of 6.78 g L^−1^.

## 1. Introduction

The untreated and indiscriminate discharges of faecal sludge into rivers, lakes and groundwater in developing and some parts of developed countries have posed considerable challenges to the environment and human health [1]. The health implications and environmental pollution ramifications are considerable. Because faecal indicator bacteria that affect human health are known to be present in high levels in faecal sludge, to make reuse efforts safe, removing these bacteria present in the waste to reach acceptable levels is necessary.

To limit the risk of faecal indicator bacterial infection, governments and researchers have introduced a set of guidelines for the proper disposal and treatment of human excreta before its use in agriculture [2]. Additionally, the faecal indicator bacteria requirements for different levels of biosolids classification (A, B and C) are defined as faecal coliforms, *Salmonella* spp., faecal *Streptococcus* and helminth eggs [3], as shown in Table 1.

As disposal of wastes has become a major problem, anaerobic digestion has been considered an alternative and sustainable waste treatment and waste management technology [4]. Anaerobic digestion not only treats waste but also generates valuable biogas [3]. However, with the intent of producing more biogas and reducing bacteria, anaerobic digestion is not enough to reduce the pathogens to meet the biosolids standards [5]. Some legislation dictates that the anaerobic digestion of waste must include pasteurisation for 60 min at 70 °C if sludge is subsequently being applied to land [6]. So, thermal pretreatment coupled with anaerobic digestion becomes essential.

However, for thermal pretreatment coupled with anaerobic digestion process, the energy requirements needed to maintain its operating conditions must be considered, such as the power consumption of mixing systems, which are directly related to the sludge rheology. The apparent viscosity is a key parameter for characterizing rheology behaviour [7].

Previous studies on the effectiveness of thermal pretreatment concentrated on improving sludge solubilisation during anaerobic digestion [8,9,10]. The anaerobic digestion process begins with hydrolysis. Insoluble organic polymers, such as carbohydrates, are broken down to soluble derivatives that become available for conversion into organic acids. Usually, the total solids (TS), volatile solids (VS), soluble chemical oxygen demand (SCOD) and soluble protein were used to estimate the degree of substrate solubilisation. However, the information about faecal indicator bacteria reduction in the pretreatment process is limited.

As such, the main objective of this study was to research the effectiveness of thermal pretreatment time and mixing on the inactivation of faecal coliform, *Salmonella* spp., and faecal *Streptococcus*, except for helminth eggs, which were not detected in raw sludge or the pretreatment process. This occurred because the sludge was obtained from the septic tank at the university, and everyone had to take special medicine to prevent helminth in China, and the environment in North China is not suitable for helminth growth [11]. Also, this study systematically evaluated the effect of sludge solubilisation under the optimal mixing parameters on inactivation kinetics, and the impact of pretreatment time and mixing.

## 2. Materials and Methods

### 2.1. Sludge Sampling and Characterization

The faecal sludge used as substrate was obtained from one of the septic tanks at the University of Science and Technology, Beijing (China). It was stored at 4 °C before digestion to reduce the influence of temperature. Its characteristics are presented in Table 2.

### 2.2. Thermal Pretreatment

The thermal pretreatment was performed at 70 °C to reduce faecal indicator bacteria, such as faecal coliform, *Salmonella* spp. and faecal *Streptococcus*. The effect of thermal treatment depends on both pretreatment time and mixing. In this work, the combined effect of pretreatment time and mixing were evaluated by submitting samples to different pretreatment times (20, 40, 60, 80, 100 and 120 min) and mixing velocity gradients (no mixing, 133, 191 and 238 s^−1^). During experiments, when the temperature of sludge increased to the pretreatment temperature, the pretreatment timing begin.

Sludge was pretreated at a thermophilic temperature of 70 °C. Four identical 1-L laboratory-scale reactors were used as anaerobic digesters. Among the four, three were stirred using a stirrer with different speeds to ensure temperature homogeneity and the fourth was not stirred. For each experiment, 1 L of sludge was used as the sample.

### 2.3. Anaerobic Digestion Reactors

**Figure 1:**
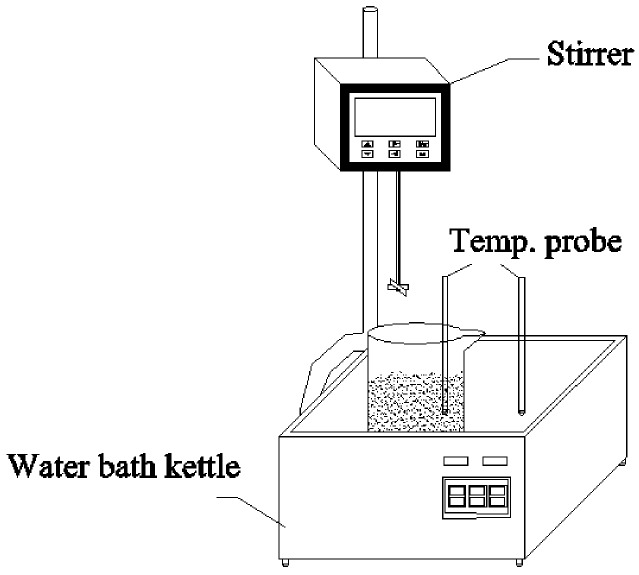
Experimental setup.

The experimental setup shown in Figure 1 consists of 1 L jars, a water bath kettle and a stirrer. The digesters were kept in a controlled thermostat water bath with a constant temperature, and the materials were mixed using a stirrer (25 W).

### 2.4. Analytical Methods

The main parameters were measured to study the faecal indicator bacteria reduction and sludge solubilisation. The chromogenic agar technique was used to test the faecal coliform, *Salmonella* spp., faecal *Streptococcus* were determined [12,13], and helminth egg was measured using the physiological saline direct smear method [14]. TS, VS, TCOD and SCOD were determined according to the APHA standard methods [15]. The pH was measured using a pH meter (HI 9125N, HANNA, Milano, Italy). TOC was analysed via the potassium dichromate volumetric method. TN was analysed with the Kjeldahl method. Protein fractions were determined by micro-bicinchoninic acid protein assays (Pierce, Rockford, IL, USA). This method, modified by Lowry et al. [16], uses a standard solution of bovine serum albumin. Soluble parameters were analysed after filtering the sludge sample through a 0.45 μm membrane filter. The apparent viscosity was tested with a rheometer (RS6000, HAAKE, Hamburg, Germany). Soluble parameters were analysed after filtering the sludge sample through a 0.45 μm membrane filter. The data are reported as the mean of three replicates. The experimental data were analysed with Microsoft Excel 2010 (Microsoft, Redmond, WA, USA) and Origin 9 (OriginLab, Northampton, PA, USA).

### 2.5. Kinetic Analysis

Concentration vs. time data obtained from the inactivation experiments were fitted to a first-order kinetic model represented as follows:

C/C_0_ = e^(−kt)^ (1)

where C is the concentration of indicator pathogen at time t, C_0_ is the concentration of indicator pathogen at time zero, and k is the first-order rate constant for inactivation at temperature T.

## 3. Results

### 3.1. Velocity Gradient

**Figure 2:**
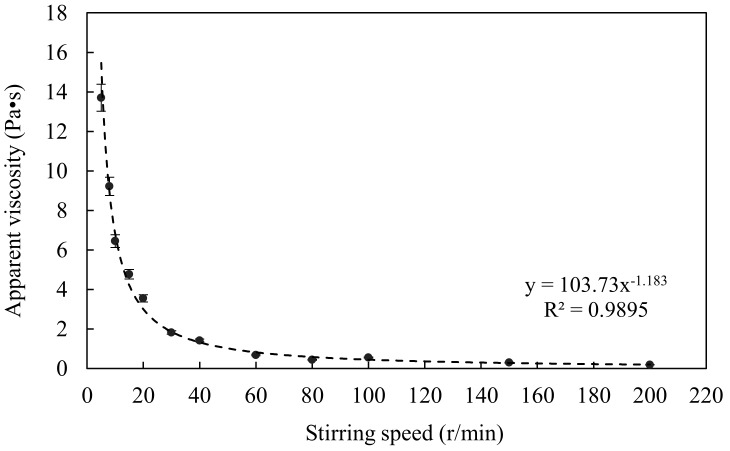
Effects of stirring speed on apparent viscosity.

In this study, the sludge was a kind of thixotropy non-Newtonian fluid. Thus, the apparent viscosity was greatly affected by the stirring speed. This experiment focused on the relationship between the stirring speed and the apparent viscosity in the same sludge at a temperature of 70 °C, with a TS concentration of 10%, and the stirring speeds were 5, 8, 10, 15, 20, 30, 40, 60, 80, 100, 150, and 200 r/min. The results in Figure 2 highlight the power exponent relationship between the apparent viscosity of sludge and the stirring speed. As the stirring speed increased, the apparent viscosity decreased. The apparent viscosities of sludge decreased significantly when the stirring speed increased from 0 to 60 r/min, whereas the apparent viscosities only slightly decreased and then gradually became constant when the stirring speed increased from 60 to 200 r/min. The apparent viscosity at stirring speeds of 40, 60, and 80 r/min is 1.43, 0.684, and 0.442 Pa·s, respectively.

Velocity gradient (G) best expresses the shear force, the higher the G value, the more intense the mixing. According to Equation (2) [17], the velocity gradient at stirring speeds of 40, 60, and 80 r/min were 133, 191, and 238 s^−1^, respectively:

(2) $$G=\sqrt{\frac{P}{\mu V}}$$

where G is the velocity gradient (s^−1^), P is the power input (W), V is the sludge volume (m^3^), and *μ* is the apparent viscosity of sludge (Pa·s).

### 3.2. Faecal Indicator Bacteria Inactivation

**Figure 3:**
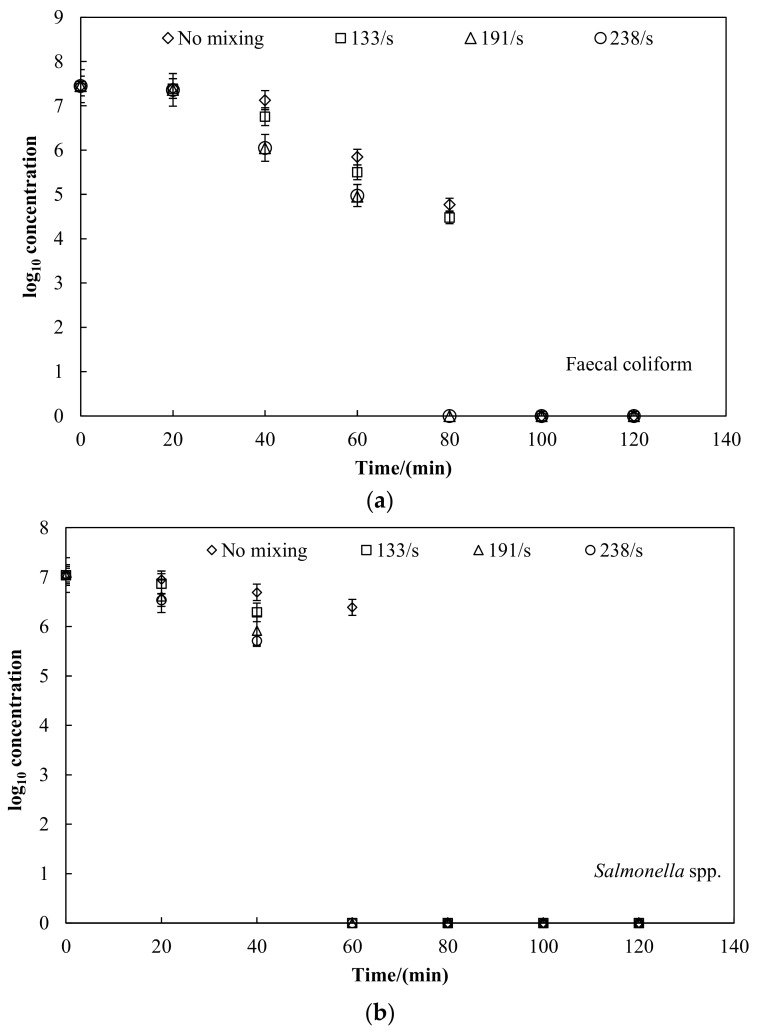

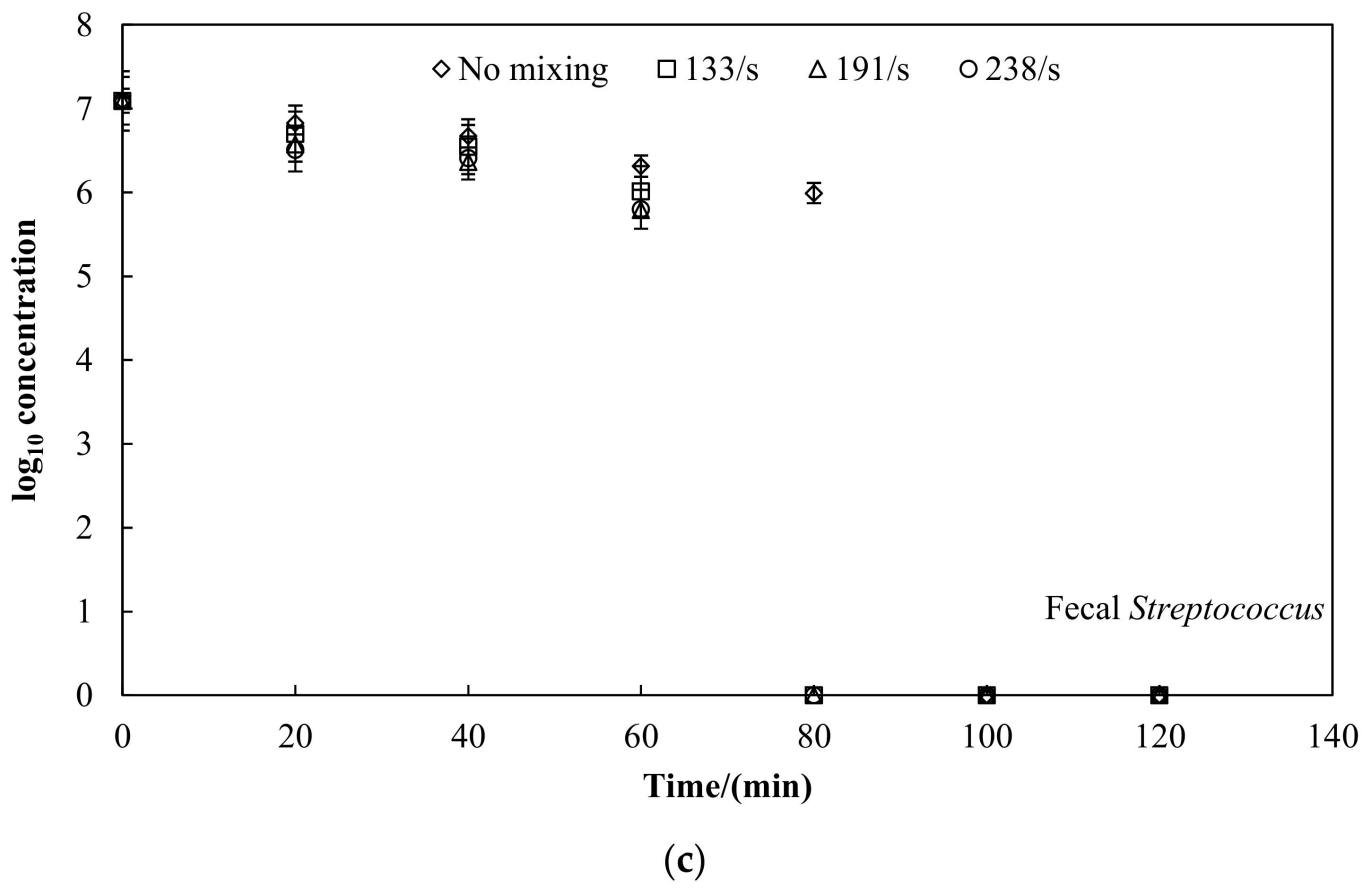
Effect of thermal (70 °C) pretreatment time on inactivation. (**a**) faecal coliform; (**b**) *Salmonella* spp.; (**c**) fecal *streptococcus*.

The highest concentrations of faecal coliform, *Salmonella* spp., and faecal *Streptococcus* were detected in raw sludge samples at 2.79 × 10^7^, 1.1 × 10^7^ and 1.24 × 10^7^ CFU/g TS, respectively. The effects of pretreatment time and velocity gradient on the inactivation of pathogens in Log10 reductions of the parameters are shown in Figure 3 [18].

The sampling schedule was developed based on the preliminary experiments that showed the faecal indicator bacteria inactivation was so rapid that the inactivation was completed within two hours at a temperature of 70 °C. Notably, some lag time existed for the inactivation [18], and in the “log10 concentration-time” figure, zero is the value of the concentration, meaning it was not detected.

As shown in Figure 3, faecal indicator bacteria were analysed at several operational stages of the pretreatment process, and the highest removals were detected during the heating process. The final concentrations of faecal coliform, *Salmonella* spp., and faecal *Streptococcus* in the reactors were all completely inactivated within two hours. Similar results were reported by Popat et al. [18] and Aitken et al. [19], who determined that the complete inactivation of the helminth ova and enteric viruses occurred within four hours at thermophilic temperature of 51–56 °C.

In this study, results indicated that a good level of hygiene is not provided if sludge is only treated for one hour at 70 °C without mixing, and the sludge does not meet the requirements for use on land without further treatment or storage. Additionally, results showed that velocity gradient had positive effect on the faecal indicator bacteria inactivation during the thermal pretreatment, as shown in Figure 3. Without mixing, faecal coliform and faecal *Streptococcus* were completely inactivated within 100 min; *Salmonella* spp. required 80 min to complete inactivation. With mixing, the pretreatment time for complete inactivation of the faecal indicator bacteria decreased. The mixing had significant effects on the *Salmonella* spp. compared to faecal coliform and *Streptococcus*. The *Salmonella* was at least as sensitive to temperature as the poliovirus vaccinal strain, so faecal coliform and *Streptococcus* appeared more resistant to thermophilic treatments [5,20].

**Figure 4:**
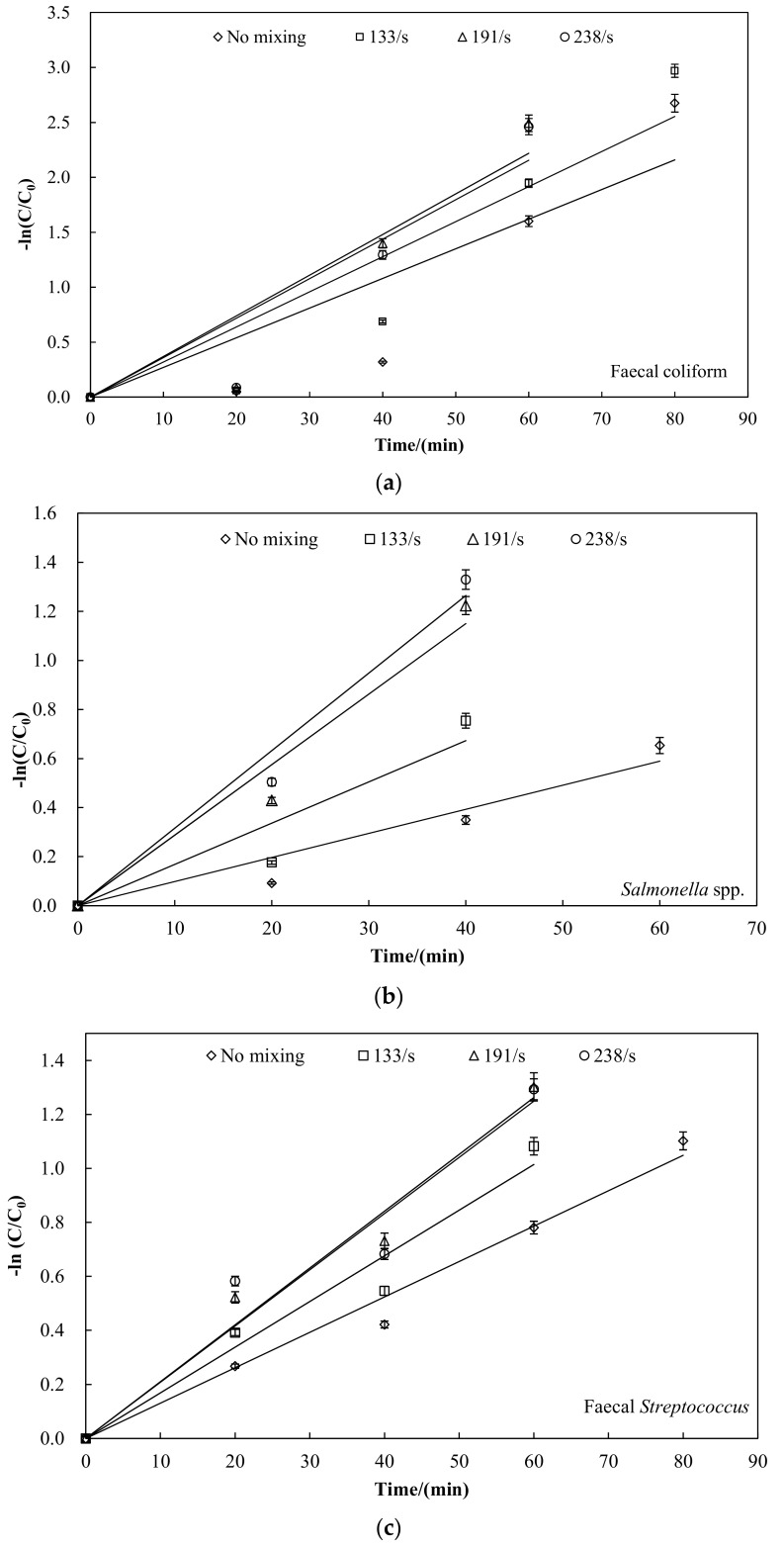
The first-order kinetics for the inactivation of faecal indicator bacteria. (**a**) faecal coliform; (**b**) *Salmonella* spp.; (**c**) fecal streptococcus.

Inactivation for faecal indicator bacteria followed first-order kinetics as indicated by the straight lines on the log-linear plots [18]. The first-order rate constants for the inactivation determined from the data collected during the inactivation experiments are shown in Figure 4, and the parameters of the first-order kinetics are shown in Table 3. For each faecal indicator bacteria the first-order rate obtained from the pretreatment with mixing was greater than with pretreatment only. Mixing had a positive effect on the faecal indicator bacteria inactivation, and reduced the pretreatment time, thus saving energy. Notably, the point of complete inactivation was not considered because zero is meaningless for Napierian logarithm. In linear fits of ln(C/C_0_) vs. time for faecal coliform, the different velocity gradients of 133, 191 and 238 s^−1^ had a related correlation coefficient (R^2^) of 0.803, 0.871, 0.878 and 0.873, respectively. Results indicated that the data obtained from the pretreatment with mixing followed first-order kinetics better than with pretreatment only. The same results were obtained for *Salmonella* spp. and faecal *Streptococcus*. With the increase in the velocity gradient, the inactivation of pathogens rate (k) increased as shown in Table 3. When the velocity gradient increased from 0 to 191 s^−1^, the inactivation rate increased significantly. When the velocity gradient increased from 191 to 238 s^−1^, the inactivation only slightly increased and then gradually became constant. Because the mixing can break off surgical incrustation and evenly distribute the solid waste, the heat transmission and energy convert efficiency are improved to a certain degree [21,22].

According to the experimental results and considering economic energy consumption, the optimal mixing conditions were obtained for our experiment: a velocity gradient of 191 s^−1^ during the thermal pretreatment.

### 3.3. Sludge Solubilisation

**Figure 5:**
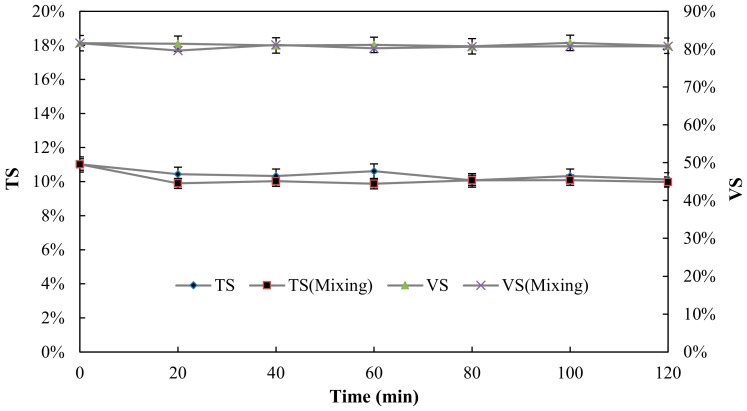
Effect of pretreatment time on TS and VS.

**Figure 6:**
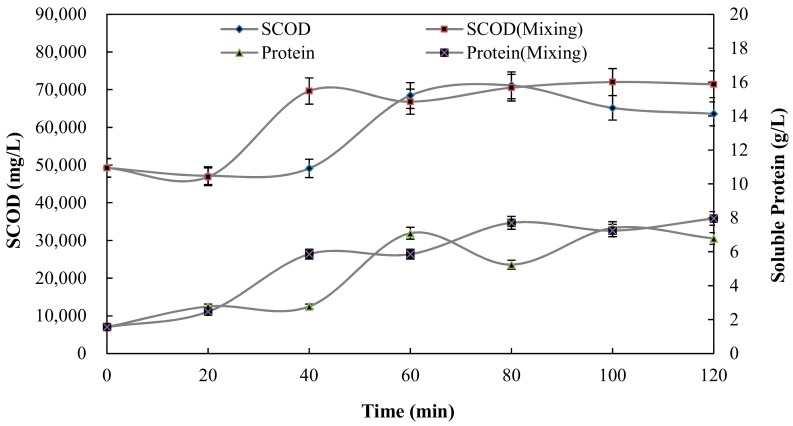
Effect of pretreatment time on SCOD and soluble protein.

The effect of thermal pretreatment on the sludge solubilisation was evaluated at the optimal mixing condition, which was a velocity gradient 191 s^−1^. Figure 5 and Figure 6 show the impact of pretreatment and mixing on sludge solubilisation. After all pretreatment, TS and VS remained almost constant compared with the untreated sludge, supporting that the organic matter has not transformed into biogas under the shorter time pretreatment. As expected, thermal pretreatment led to sludge solubilisation by heating the sludge from the pretreatment reactor operated at 70 °C.

The degree of substrate solubilisation can be estimated from the SCOD and soluble protein. The SCOD and soluble protein values were several times higher in the pretreated sludge than in the raw sludge (SCOD 49,250 mg/L, soluble protein 1.56 g/L) (as shown in Figure 6), confirming previously-reported significant solubilisation effects of heating on sewage sludge [23,24]. Simultaneously, for combined thermal pretreatment and mixing, SCOD (71,430 mg/L) and soluble protein (7.96 g/L) were higher than sum of the thermal pretreatment values (SCOD was 63,600 mg/L, soluble protein was 6.78 g/L), indicating a clear synergetic effect. Therefore, thermal pretreatment had weakened the cell walls and led to the release of more soluble organics when combined with mixing [21].

The high release of colloidal particles in solution by the hydrolysis step was reported to decrease the ability of the digested sludge to filter [25]. A large amount of dispersed charged fine particles and biocolloids were also reported to be released by the biological hydrolysis of the sludge, which consequently caused an increase in the particles with a colloidal charge such as the SCOD [26]. The degradation of sludge solids during the anaerobic digestion process generated changes in the physical-chemical properties of the floc, causing the release of its intracellular components, changes in its morphology, and an increase in its true colloidal content [10].

Protein content in sludge is generally composed of three parts: cell, bound and solubility [27], among which the soluble protein represents the protein in the aqueous phase of the sludge. As shown in Figure 6, thermal pretreatment had a significant impact on the protein solubilisation, as the soluble protein increased several fold relative to the control after the pretreatment. Additionally, further improvement in sludge solubilisation occurred by mixing doses with the thermal pretreatment compared with thermal pretreatment alone [28].

## 4. Conclusions

The combination of thermal pretreatment (70 °C) and mixing was better than thermal pretreatment alone for improving the faecal indicator bacteria inactivation. For combined thermal pretreatment and mixing, 80 min was sufficient to completely inactivate the faecal indicator bacteria to fulfil the requirements for use on land.

The faecal indicator bacteria inactivation kinetics can be predicted by the first-order kinetics model. With the increase in mixing speed, the inactivation rate increased. The optimal mixing condition was the velocity gradient of 191 s^−1^. At the optimal mixing conditions, the thermal pretreatment had a positive effect on the sludge solubilisation, SCOD (71,430 mg/L) and soluble protein (7.96 g/L) were higher than the sum of the values for thermal pretreatment alone (SCOD was 63,600 mg/L, soluble protein was 6.78 g/L).

## Figures and Tables

**Table 1:** Requirements for biosolids treatment for different classification levels.

Level	Faecal Indicator Bacteria	US-EPA/625/R92	Mexico-NOM-004-SEMARNAT
A	faecal coliforms	<10^3^ CFU/g TS	<10^3^ CFU/g TS
	*Salmonella* spp.	<3 CFU/g TS	<3 CFU/g TS
	faecal *Streptococcus*	<10^3^ CFU/g TS	--
	helminth eggs	<1 egg/g TS	<1 egg/g TS
B	faecal indicator bacteria	<2 × 10^6^ CFU/g TS	<10^3^ CFU/g TS
	faecal coliforms	<3 CFU/g TS	<3 CFU/g TS
	*Salmonella* spp.	<10^6^ CFU/g TS	--
	faecal *Streptococcus*	<1 egg/g TS	<10 egg/g TS
C	faecal indicator bacteria	--	<2 × 10^3^ CFU/g TS
	faecal coliforms	--	<300 CFU/g TS
	*Salmonella* spp.	--	--
	faecal *Streptococcus*	--	<35 egg/g TS

**Table 2:** Initial characterization of the faecal sludge.

Analytical Parameters	Faecal Sludge
Total nitrogen (TN) (g L^−1^)	0.209 ± 0.018
Total organic carbon (TOC) (g L^−1^)	5.02 ± 0.27
Total solid (TS)	11 ± 0.7%
Volatile solids (VS)	81.6 ± 1.6%
pH	6.9 ± 0.25
Total chemical oxygen demand (TCOD) (mg L^−1)^	101,300 ± 100
Soluble chemical oxygen demand (SCOD) (mg L^−1^)	49,250 ± 60
Protein (g L^−1^)	24.1 ± 2.3
Soluble protein (g L^−1^)	1.56 ± 0.12
Faecal coliform (CFU g^−1^ (TS))	(2.79 ± 0.04) × 10^7^
*Salmonella* spp. (CFU g^−1^ (TS))	(1.10 ± 0.02) × 10^7^
Faecal *Streptococcus* (CFU g^−1^ (TS))	(1.24 ± 0.02) × 10^7^
Helminth eggs (Egg g^−1^ (TS))	0

**Table 3:** Parameters of the first-order kinetics during thermal pretreatment.

Faecal Indicator Bacteria	Mixing Speed/(r·min^−1^)	First-Order Kinetics	
		k	R^2^
faecal coliform	0	0.027	0.803
	40	0.031	0.871
	60	0.037	0.878
	80	0.035	0.873
*Salmonella* spp.	0	0.009	0.894
	40	0.016	0.905
	60	0.029	0.965
	80	0.030	0.977
faecal *Streptococcus*	0	0.013	0.932
	40	0.016	0.959
	60	0.021	0.972
	80	0.020	0.968
